# COVID-19 in Adult Patients with Hematological Malignancies—Lessons Learned after Three Years of Pandemic

**DOI:** 10.3390/biology12040545

**Published:** 2023-04-03

**Authors:** Iwona Hus, Agnieszka Szymczyk, Joanna Mańko, Joanna Drozd-Sokołowska

**Affiliations:** 1 Department of Hematology, National Medical Institute of the Ministry of Interior and Administration, 137 Wołoska Str., 02-507 Warsaw, Poland; 2 Department of Clinical Transplantology, Medical University of Lublin, 7 Chodźki Str., 20-093 Lublin, Poland; 3 Department of Hematology, Oncology and Internal Medicine, Medical University of Warsaw, 1a Banacha Str., 02-097 Warsaw, Poland

**Keywords:** SARS-CoV-2, COVID-19, treatment, prophylaxis, hematological malignancies

## Abstract

**Simple Summary:**

The COVID-19 pandemic significantly affected the clinical outcomes and management of patients with hematological malignancies, who are especially vulnerable to infections. At the time of the pandemic outbreak, when a highly virulent wild-type strain of SARS-CoV-2 (B.1) was responsible for the majority of infections, mortality rates among hematology-oncology patients was high. As no specific prevention or treatment was available at the time, patients were strictly advised to wear masks and practice physical distancing and hand hygiene. When anti-cancer treatment was needed, protocols with oral drugs on an outpatient basis were preferred to avoid hospitalization whenever possible. Despite this, many hemato-oncological patients died from COVID-19, secondary bacterial or fungal infections as well as from delayed or suboptimal antineoplastic treatment. Less virulent viral variants contributed to reducing the problem with COVID-19 in the general population, but patients with hematologic malignancies are still at high risk of suffering from SARS-CoV-2 or COVID-19 infection with a severe or long clinical course. These patients are advised to get vaccinated, wear a face mask and avoid poorly ventilated or crowded places. CDC guidelines recommend starting antiviral treatment as soon as possible in case of a positive SARS-CoV-2 test, i.e., within 5 days of the first symptoms.

**Abstract:**

The COVID-19 pandemic is undoubtedly the most difficult health challenge of the 21st century with more than 600 million laboratory-confirmed SARS-CoV-2 infections and over 6.5 million deaths worldwide. The coronavirus pandemic contributed to rapid development of mRNA vaccines, which, along with new antiviral drugs, have been the subject of extensive research for many decades. Nevertheless, elderly, multi-morbid and immunocompromised patients continue to face a more severe clinical course and a higher risk of death from COVID-19, even now that the risk of COVID-19 in the general population is significantly reduced due to the introduction of global vaccination strategies. In this paper, we present the mechanisms of increased susceptibility to infectious complications and the evolution of the clinical course of COVID-19 in patients with hematological malignancies, taking into account the mutation of the virus and the introduction of vaccines and new antiviral drugs. We also present current recommendations for prophylactic and therapeutic management in patients with hematological malignancies.

## 1. Introduction

Hematological malignancies (HMs) are diverse group of neoplastic diseases deriving from myeloid or lymphoid cells. Depending on the type of malignancy as well as on the treatment used, hematological malignancies are associated with different types of immune disorders. Immune defects are much more pronounced in patients with lymphoid malignancies, especially chronic lymphocytic leukemia (CLL) or multiple myeloma (MM) [1]. In both of these diseases, complex disturbances in the number and function of various immune cell populations result in an increased susceptibility to various infectious complications. Additional factors predisposing to infection include elderly age (>65 years) and comorbidities such as diabetes, renal insufficiency or cardiac failure [2,3,4,5,6]. Many therapies commonly used in the treatment of lymphomas, such as cytostatic agents (fludarabine and bendamustine) [7,8,9], B-cell-depleting therapies (anti-CD20 monoclonal antibodies) [10,11], Bruton kinase (BTK) inhibitors [12,13] and chimeric antigen receptor (CAR) T-cell therapy [14,15], might cause severe and long-lasting immunosuppression. According to a report by Spanjaart et al. [16] on behalf of European Bone Marrow Transplantation (EBMT) Infectious Diseases Working Party and the European Hematology Association (EHA) Lymphoma Group [16], mortality in patients treated with CAR-T was as high as 41.1%. Immune deficiencies are much less pronounced in patients with chronic myeloid malignancies, such as chronic myeloid leukemia (CML) or Ph-negative myeloproliferative neoplasms (MPN). Therapies such as BCR-ABL tyrosine kinase inhibitors or alkylating agents do not induce immunosuppression [17,18]; however, JAK-2 inhibitors used in patients with MPN suppress the immune system, and their primary side effect is susceptibility to infections [19,20,21]. In turn, patients with acute leukemias are at high risk of infections due to severe qualitative and quantitative deficits in granulocytes or lymphocytes caused by the disease itself as well as the chemotherapy used [22,23]. Autologous (ASCT) and allogeneic (allo-SCT) stem cell transplantation is always associated with immune cell depletion. Furthermore, in patients that undergo allo-SCT, immunosuppression might be further increased by the agents used in the prophylaxis and/or treatment of graft versus host disease (GvHD) [24,25]. Although the response to vaccination is lower than in the healthy population due to combined immunodeficiencies [26,27,28], vaccination is the recommended infection prevention strategy for all patients with hematological malignancies [29,30].

The COVID-19 pandemic, caused by severe acute respiratory syndrome coronavirus 2 (SARS-CoV-2), began in Wuhan, China, in November 2019 and by now (data from 25 January 2023) has affected 664,618,938 individuals and resulted in 6,722,949 deaths worldwide [31]. The COVID-19 pandemic spanned several successive phases, with the highest morbidity and mortality rates reported in 2020 (https://ourworldindata.org/mortality-risk-covid). The risk factors for a severe clinical course of COVID-19 include old age, male gender and comorbidities such as diabetes, hypertension, chronic lung, liver, heart and kidney diseases, obesity and neoplasms, especially hematological malignancies [32,33,34]. Although the COVID-19 pandemic is far from over, morbidity and mortality rates have decreased significantly thanks to a number of factors, most notably the introduction of vaccines and new antivirals [35,36]. Mutations of the SARS-CoV-2 virus, leading to the emergence of variants with different transmissibility and virulence, contribute to significant variability in the clinical picture of infections during the pandemic. Nevertheless, COVID-19 still remains a health problem in vulnerable populations such as patients with hematological malignancies. In this review, we summarize how the COVID-19 pandemic influenced the outcomes and management in patients with HMs as well as the current approach to COVID-19 prophylaxis and treatment in this population.

## 2. SARS-CoV-2 Structure and Variants

On the 11th of February 2020, the International Committee on Taxonomy of Viruses formally renamed “2019 novel coronavirus” (2019-nCoV) as severe acute respiratory syndrome coronavirus 2 (SARS-CoV-2), and the WHO formally named the viral illness Coronavirus Disease 2019 (COVID-19) [37].

SARS-CoV-2 is an enveloped RNA virus belonging to the Betacoronavirus genus in the Coronaviridae family. Each SARS-CoV-2 virion is generally spherical, with a diameter of 60–140 nm. The virus is surrounded by distinct spikes 9–12 nm long, giving it a solar-corona-like appearance, hence its name. SARS-CoV-2 consists of positive-sense single-stranded RNA (+ssRNA) and viral structural proteins. The (+ssRNA) is built with about 30,000 base pairs and is used directly as a template for protein synthesis (of structural, non-structural and accessory proteins). Replication of the genetic material of the virus consists of transcribing the genome into a strand of (−)RNA, which serves as a template for the synthesis of new (+)RNA genomes [38].

SARS-CoV-2 contains the following four structural proteins:

- S (spike)—fusion protein or surface glycoprotein—responsible for interaction with the receptor on the surface of host cells;
- E (envelope)—coat protein—responsible for the formation of virions;
- M (membrane)—membrane or membrane glycoprotein—the main protein of the virus matrix;
- N (nucleocapsid)—a nucleocapsid protein—protecting a large RNA molecule and participating in the modification of cellular processes and virus replication.

The fundamental function of the N protein is to package the viral genome RNA into a long helical ribonucleocapsid (RNP) complex, and the S, E and M proteins form the viral envelope. The S protein is responsible for binding to the membrane of the host cells. The S-glycoprotein is functionally differentiated into S1 and S2 subunits. The S1 subunit mediates binding to the host cell surface receptor, and the S2 subunit mediates fusion with the cell membrane; then the virus enters the host cell by endocytosis [39,40,41].

An immanent feature of all viruses, including SARS-CoV-2, is their ability to create numerous changes in their genetic material, which allows for adaptation to variable environments and escape from host defense mechanisms. The accumulation of SARS-CoV-2 virus mutations resulting from virus replication is therefore a completely natural phenomenon, and the occurrence of new SARS-CoV-2 variants over the three years of the pandemic highlights one of the main challenges facing modern medicine. In 2020, the WHO introduced designations of new SARS-CoV-2 strains as variants of concern (VOCs) and variants of interest (VOIs) [37]. In particular, VOCs have been defined as variants with increased transmission, greater severity of the clinical course of the disease, markedly reduced neutralization by antibodies and thus reduced response to treatment and vaccines. VOIs include variants with mutations related to receptor binding, reduced treatment efficacy and neutralization by antibodies and potentially increased disease severity and/or transmission [37]. In order to standardize the names of new SARS-CoV-2 strains, the WHO introduced successive letters of the Greek alphabet to name novel virus variants [38].

COVID-19 mainly affects the respiratory system, with a clinical picture ranging from mild flu-like symptoms, such as fever, cough, sore throat, rhinitis and fatigue, to life-threatening symptoms of severe acute respiratory syndrome (SARS) [42,43,44] requiring intensive care unit (ICU) admission. Other systems may also be involved, such as the gastrointestinal tract, cardiovascular system or urinary system. On average, the first symptoms appear 5–6 days after exposure to the virus, and patients with mild symptoms usually recover within 2 weeks; in severe cases, recovery may take up to 6 weeks [44].

Increased replication in host cells, resulting in greater viral load and dysregulation of innate and adaptive immune responses, is believed to be the most important mechanism contributing to the increased virulence and risk of death from SARS-CoV-2 infection compared to other viruses. Additional mechanisms of pathology specific to this virus, such as dysregulation of the renin–angiotensin–aldosterone system (RAAS), systemic hyper-inflammatory response to infection and macro- and microvascular thrombosis, contribute to the wide clinical variability of infection in the human population [40].

## 3. Treatment of Patients with COVID-19

When deciding on the treatment method in patients with COVID-19, one should take into account not only the clinical condition and stage of the disease but also risk factors for severe course of the disease [45]. The use of antivirals is recommended even in asymptomatic or mildly symptomatic patients with hematological malignancies, especially those undergoing or within 12 months of completion of B-cell-depletion therapy or in the early period after allo-SCT, where immunosuppressants are used for the prevention or treatment of GvHD [46,47]. The use of antiviral drugs brings the greatest benefits during the period of intensive viral replication, while in the second phase of the disease, when the immune system is overstimulated, their use usually does not bring satisfactory results [45].

**Figure 1:**
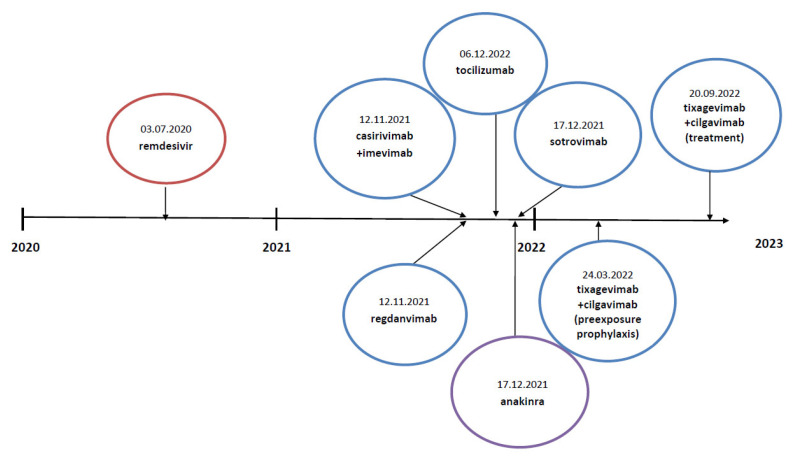
History of drugs for COVID-19 treatment registered by the European Medicines Agency (EMA).

During the COVID-19 pandemic, the effectiveness of many drugs was assessed, such as chloroquine and hydroxychloroquine, azithromycin, doxycycline, favipiravir, lopinavir/ritonavir, oseltamivir, amantadine, rimantadine, zanamivir, acyclovir, ivermectin, colchicine and intravenous immunoglobulins (IVIg). However, due to the lack of effectiveness evidence, they have not been used in the therapy of SARS-CoV-2 infection. Other drugs (e.g., casirivimab/imdevimab) have ceased to play a significant role in the treatment due to the emerging new virus variants [48]. Figure 1 presents drugs for COVID-19 registered in subsequent years by the European Medicines Agency (EMA) [49]. Clinical trials evaluating the efficacy of new therapies are still ongoing [48]. The most important drugs registered and currently used for the treatment of COVID-19 are characterized below. The most important studies on the effectiveness of antiviral drug used for the treatment of COVID-19 in patients with hematological malignancies are presented in Table 1.

## 4. Convalescent Plasma

At the beginning of the pandemic, when COVID-19 treatment options were intensively sought, it seemed that the use of convalescent plasma could be an easily and quickly available therapeutic option. Passive transmission of antibodies directed against SARS-CoV-2 was expected to alleviate the course of infection [48,60]. The first observational studies and case series suggested a beneficial effect of convalescent plasma administered in the early phase of viral load during SARS-CoV-2 infection, but these observations have not been confirmed in large randomized clinical trials [61,62]. In the RECOVERY study, which compared the use of convalescent plasma in combination with standard of care with standard of care alone, there were no significant differences in recovery time, 28-day mortality rate and percentage of patients requiring mechanical ventilation [63]. Similar conclusions were drawn from the REMAP-CAP study [64]. A study by Bégin et al. also showed no significant differences in mortality or the need for ventilation therapy [65]; however, Weinbergerova et al. indicated the effectiveness of convalescent plasma in patients with HM [57]. Currently, the routine use of convalescent plasma is not recommended, but it may be an option in selected immunocompromised patients, including those with hematological malignancies [47,66].

## 5. Glucocorticoids

The use of glucocorticoids may be beneficial in the disease phase with excessive activation of the immune system due to their anti-inflammatory effect. This was confirmed by numerous studies conducted in patients with severe COVID-19 as well as in meta-analyses. It has been proven that long-term glucocorticoids therapy might be associated with side effects, such as carbohydrate metabolism disorders, hypertension, cataracts, infections, fluid retention, increased risk of gastrointestinal bleeding or hypernatremia. Their short-term use, however, also brings significant clinical benefits [48,60,67,68]. In the RECOVERY study, the use of dexamethasone reduced mortality in both mechanically ventilated patients (29.3% vs. 41.4%) and those requiring oxygen therapy (23.3% vs. 26.2%) [67]. The use of glucocorticoids has not been shown to be of benefit in patients who did not require ventilatory support. If dexamethasone is not available, another equivalent glucocorticoid may be considered, i.e., prednisone 40 mg/day, methylprednisolone 32 mg/day or hydrocortisone 160 mg/day. Clinical data on the use of glucocorticoids other than dexamethasone are limited. Increasing the daily dose of dexamethasone to 12 mg/day is not recommended due to lack of clinical benefit [45,48,68,69]. Dexamethasone is now recommended for patients with severe COVID-19 who require oxygen or ventilator therapy [48,60,68,69].

## 6. Tocilizumab (RoActemra)

Tocilizumab is a recombinant, humanized IgG1k monoclonal antibody directed against the IL-6 receptor (IL-6R) [48,70]. Its effectiveness in COVID-19 patients with hyperinflammation and cytokine storm was confirmed in the RECOVERY study. Patients with CRP ≥ 75 mg/L and oxygen saturation < 92% treated with tocilizumab had a lower 28-day mortality compared to placebo (29% vs. 33%, *p* = 0.007) [71]. In the REMAP-CAP study in patients with severe COVID-19, mortality was also lower in the tocilizumab group compared to placebo (28% vs. 35.8%, respectively) [72]. The EMPACTA study demonstrated superiority of tocilizumab in terms of 28-day mortality and mechanical ventilation rate, but it did not show any improvement in total mortality rate [73]. The TOCIBRAS study was prematurely ended by the data monitoring committee after enrollment of 129 patients due to an increased number of deaths at day 15 in patients receiving tocilizumab (18 patients in the tocilizumab group died or received mechanical ventilation compared to 13 patients in the placebo group) [74]. Hermine et al. also showed that 28-day mortality in the tocilizumab group was similar to standard of care (7 patients vs. 8 patients, respectively) [75]. Despite this, tocilizumab is still recommended for patients with moderate and severe COVID-19 with cytokine storm, predominantly in combination with dexamethasone [47,48,49].

## 7. Remdesivir (Veklury)

Remdesivir is a nucleotide analog that inhibits viral RNA-dependent RNA polymerase [48,60,66], and it has broad-spectrum activity against RNA viruses (including SARS-CoV-1 and MERS viruses) [76,77]. It was first used to treat SARS-CoV-2 infection in a 35-year-old man with hypertriglyceridemia in the United States, which in turn initiated wider attempts to use it in clinical practice [78]. In subsequent prospective studies, Grein et al. [79] and Antinori et al. [80] confirmed the efficacy of remdesivir in the treatment of patients requiring oxygen therapy.

Beigel et. al. [81] demonstrated in a randomized, double-blind clinical trial shortened recovery time both in the entire study group (recovery time 10 days in the remdesivir arm vs. 15 days in the placebo group, *p* < 0.001) and in the group of patients requiring oxygen therapy (11 vs. 18 days). The remdesivir group also showed a trend towards a reduction in mortality (11.4% vs. 15.2%) and less frequent use of mechanical ventilation or ECMO (13% vs. 23%) [81]. In a phase III clinical trial, Goldman et al. [82] compared the safety and efficacy of remdesivir administered for 10 days or 5 days in patients requiring passive oxygen therapy who were not mechanically ventilated. There were no significant differences between the two arms [82].

The World Health Organization initiated the SOLIDARITY study, evaluating the effectiveness of other therapies such as hydroxychloroquine, interferon-β-1a and lopinavir/ritonavir. No significant benefits were demonstrated in the patients receiving remdesivir compared to the control arm in terms of the analyzed endpoints: 28-day in-hospital mortality (10.9% vs. 11.2%), the need for mechanical ventilation (11.9% vs. 11.5%) and the percentage of patients hospitalized on day 21 (9% vs. 8%) [83]. The results of the DisCoVeRy study did not confirm a reduction in mortality in patients with moderate COVID-19 treated with remdesivir; however, the authors pointed out limitations of this study (an open-label, non-placebo-controlled clinical trial with no information on the supportive care and the duration of symptoms from COVID-19 diagnosis to therapy initiation [84]). In the PINETREE study, early use of remdesivir reduced the frequency of hospitalization and the risk of death in a small group of immunocompromised patients (*p* = 0.008) [85]. Remdesivir was also effective in patients with HMs [55,56,57,86,87].

Finally, remdesivir was approved for the treatment of coronavirus disease 2019 (COVID-19) in adults and adolescents (aged 12 years and older with a body weight of at least 40 kg) with pneumonia requiring supplemental oxygen and who were at increased risk of progression to severe disease [49]. The main contraindication to the use of remdesivir is renal impairment with a GFR <30 mL/min. Liver function should also be monitored during therapy [48,66].

## 8. Molnupiravir (Lagevrio)

Molnupiravir is an oral prodrug that is metabolized to the ribonucleoside analogue N-hydroxycytidine, which is then phosphorylated to form the pharmacologically active ribonucleoside triphosphate acting via a mechanism known as viral error catastrophe [88]. Its effectiveness was demonstrated in the MOVe-OUT study, which recruited patients with at least one risk factor for a severe course of COVID-19, in whom the first symptoms of the disease occurred within 5 days of inclusion in the study. Hospitalization rate was significantly lower in the subgroup of patients receiving molnupiravir within 3 days of symptom onset (6.8% vs. 9.7%). There was no increase in the incidence of adverse events during treatment [89]. In a phase 2a clinical trial, Fisher et al. [90] showed significantly shorter viral clearance time (14 vs. 15 days, *p* = 0.013) in non-hospitalized patients with mild to moderate COVID-19. In the molnupiravir 800 mg/day group, the proportion of patients who were viral positive was lower on day three of treatment compared to those on placebo (1.9% vs. 16.7%; *p* = 0.016). There was no increase in the incidence of adverse events in the molnupiravir 800 mg/day group compared to 200 mg/day and 600 mg/day groups [90]. The efficacy of molnupiravir and its favorable safety profile were also confirmed in patients with HM [58]. Other publications indicated that the use of molnupiravir in patients with severe or moderate COVID-19 did not bring clinical effects [91]. Molnupiravir is approved for use in patients with COVID-19 who do not require oxygen therapy and are at increased risk of disease progression to a severe form [92].

## 9. Nirmatrelvir/Ritonavir (Paxlovid)

Nirmatrelvir is a SARS-CoV-2 protease inhibitor 3CLpro that inhibits viral replication at the proteolytic stage [93]. The addition of ritonavir slows down the metabolism and helps maintain a high concentration of the drug, which translates into longer action [47,66,93]. The efficacy of nirmatrelvir/ritonavir was confirmed in a phase II/III study by Hammond et al. [94]. The study was conducted in a group of 2246 patients with mild or moderate COVID-19 in whom symptoms occurred no later than 5 days before randomization and who had at least one risk factor for a severe course of infection. There was an 89.1% relative risk of hospitalization (9 vs. 67) and death (0 vs. 12) from any cause compared to the placebo group. A favorable safety profile of the drug was demonstrated, with the most common adverse reactions including dysgeusia (5.6%), diarrhea (3.1%) and nausea and vomiting (1.1%) [94]. The drug is recommended for use in patients with mild or moderate COVID-19 who do not require oxygen therapy and with risk factors for a severe course of the disease [48].

## 10. Sotrovimab (Xevudy)

Sotrovimab is an IgG1 monoclonal antibody that binds to a highly conserved epitope of the S protein of SARS-CoV-2 [49]. Its effectiveness was assessed in the COMET-IC study, which recruited unvaccinated patients with mild or moderate COVID-19 who did not require oxygen therapy had at least one risk factor for severe COVID-19 and whose clinical symptoms of infection occurred within 5 days before randomization. Sotrovimab significantly reduced the risk of progression to severe or critical COVID-19 (*p* < 0.001) [95]; the effectiveness of the drug was also reported in patients with HMs [96]. Based on this study, sotravimab was approved by the EMA and FDA for the treatment of patients who do not require oxygen therapy and are at increased risk of progression to severe COVID-19. Unfortunately, its activity against the SARS-CoV-2 variant BA.2 is significantly lower than against the wild strain [49]. On 5 April 2022, the FDA announced that it is no longer authorized to treat COVID-19 in any U.S. region due to increases in the proportion of COVID-19 cases caused by the Omicron BA.2 sub-variant [97]. Dean et al. reported one case of a successfully treated patient with acute myeloid leukemia and mild COVID-19 infection, who received antiviral therapy with remdesivir and sotrovimab [98]

## 11. Tixagevimab/Cilgavimab (Evusheld)

Evusheld contains tixagevimab in combination with cilgavimab, two long-acting recombinant IgG1κ human monoclonal antibodies [49,99]. The EMA and FDA approved tixagevimab/cilgavimab based on the results of the TACLE study, which recruited non-hospitalized, unvaccinated patients with confirmed COVID-19 infection diagnosed within 3 days of randomization. Death from any cause or COVID-related deaths were reported in eighteen patients from the study group and thirty-seven patients from the placebo group (4% vs. 9%; *p* = 0.0096). There were six COVID-19-related deaths in the placebo group and three in the tixagevimab/cilgavimab group [100]. Evusheld can be given to adults and adolescents > 12 years of age and weighing more than 40 kg. Tixagevimab/cilgavimab is used in patients who do not require oxygen therapy and are at risk of severe course of COVID-19 [49]. On 26 January 2023, the FDA revised the Emergency Use Authorization (EUA) for Evusheld to limit its use for pre-exposure COVID-19 prophylaxis in the US until further notice due to the persistently high frequency of circulating SARS-CoV-2 variants against which Evusheld did not show in vitro neutralizing activity [101].

## 12. Prophylaxis of SARS-COV-2 Infection

### 12.1. Vaccines

Since 2020, the development of a vaccine against SARs-CoV-2 has been a huge challenge for the entire medical community. On 2 December 2020, the United Kingdom’s Medicines and Healthcare products Regulatory Agency issued temporary approval for the Pfizer–BioNTech vaccine, becoming the first country in the Western world to approve the use of any COVID-19 vaccine. By 21 December 2020, many countries and the European Union had approved the Pfizer–BioNTech COVID-19 vaccine. In the following months, other pharmaceutical companies obtained FDA and EMA approval for new SARS-CoV2 vaccines [102,103].

Technology using the mRNA platform has been already developed for many years in the production of viral vaccines as well as in cancer therapies. However, it was used for the first time in vaccine production on such a massive scale. mRNA vaccines against COVID-19 consist of messenger ribonucleic acid encoding the S protein (spike) of the SARS-CoV-2 virus encapsulated in lipid nanoparticles. The capsule has a protective and transport function, helping to penetrate into the cell by overcoming the cell membrane barrier. On the basis of mRNA, S protein is synthesized in the host cell, inducing both humoral (neutralizing antibodies) and cellular responses (cytotoxic T-cells) [104]. Vaccines approved in the European Union that can be used both as primary vaccination and booster are presented in Table 2 [105].

It is recommended that eligible persons be vaccinated with one of these vaccines. If availability is not an issue, it is suggested to use an mRNA vaccine (BNT162b2 or mRNA-1273) or NVX-CoV2373. Extensive data supporting the use of mRNA vaccines have accumulated since their availability. Less data on safety and efficacy of NVX-CoV2373 are available, but it is also highly effective and may be an attractive option for individuals with concerns about the novelty of the mRNA vaccine platform. The formulations for children are age-specific and differ from that used in older individuals [102].

Vaccination against SARS-CoV-2 of healthy people has been shown to be highly effective in preventing COVID-19, reducing the severity of infection, including the need for hospitalization, and reducing the mortality rate of infected patients, which significantly contributed to changing the course of the COVID-19 pandemic in individual countries.

Data on COVID-19 vaccination in patients with HMs indicated their limited effectiveness due to impaired immunity resulting from underlying disease and its treatment. Information on the safety and the efficacy of vaccines in HM patients is based mostly on retrospective data, as cancer patients were excluded from most clinical trials or constituted a small proportion of the study populations [112]. Results of the pooled analysis of 22 controlled studies including 3187 patients with HMs showed that the seroconversion rate after the first dose of COVID-19 vaccine was 33.3% compared to 74.9% in the controls [113]. The seroconversion rate increased after the second dose, although it was still lower in HM patients (65.3% vs. 97.8%) mainly due to low seroconversion rate in patients with CLL and B-cell leukemia/lymphoma treated with anti-CD20 antibodies or BTK inhibitors [113].

The data on the incidence and outcomes of COVID-19 in patients with HMs from 42 countries were collected in an international observational study (Epidemiology of COVID-19 Infection in Patients with Hematological Malignancies: A European Hematology Association Survey- EPICOVIDEHA). Over 80% of patients in this registry were diagnosed with lymphoid malignancies (CLL, NHL and MM), and 68.1% were treated for HM. The majority of patients (77%) were vaccinated against COVID-19. Serological response to vaccination was analyzed in 35.4% fully vaccinated patients. In 32.5% of analyzed patients, an IgG antibody response to the vaccine was noted [106].

In the UK PROSECO study (prospective observational study evaluating COVID-19 vaccine responses), no humoral response was detected after two or three vaccine doses in more than a half of patients with B-cell malignancies receiving anticancer treatment. Furthermore, there were no anti-SARS-CoV-2 antibodies after the full vaccination schedule in 60% of patients receiving anti-CD20 therapies within the last 12 months [114].

The EPICOVIDEHA study was updated with assessing of breakthrough COVID-19 in 1548 HM patients, including 76% with lymphoid malignancies [107]. The majority of patients (89%) received at least two doses of the COVID-19 vaccine. Omicron was the dominating variant of the SARS-CoV-2 virus (68.7% of cases). Specific treatment for COVID-19 was given to 59% of patients. In patients infected with the Omicron variant, mortality was 7.9% and was significantly lower compared to the pre-vaccination period (31%). Treatment with monoclonal antibodies was associated with lower mortality even in patients with severe or critical COVID-19 [107].

In September 2022, EMA’s human medicines committee recommended authorizing an adapted bivalent vaccine targeting the Omicron subvariants BA.4 and BA.5 in addition to the original strain of SARS-CoV-2. Comirnaty Original/Omicron BA.4-5 (Pfizer/BioNTech) and Spikevax bivalent Original/Omicron BA.4-5 (Moderna) are intended for use in individuals 12 years of age and older who have received at least a primary course of vaccination against COVID-19 [105]. They are expected to extend protection against different variants and help maintain optimum levels of protection against COVID-19 as the virus evolves.

### 12.2. Tixagevimab/Cilgavimab

The efficacy of Evusheld in pre-exposure prophylaxis was proven in the PROVENT study, which was conducted in 5197 patients who had increased risk of an inadequate response to vaccination, had a high risk of infection or both. COVID-19 infection was diagnosed in eight patients from the experimental group and seventeen from the placebo group (0.2% vs. 1.0%; *p* < 0.001). Five cases of severe or critical COVID-19 and two COVID-19-related deaths were reported only in the placebo group [115]. Ocon et al. also confirmed the effectiveness of Evusheld in pre-exposure prophylaxis in patients with HMs with a favorable safety profile [116].

### 12.3. COVID-19 in Patients with Hematological Malignancies during the First Phases of Pandemic (before Introduction of the Prophylaxis Strategies)

The first reports on the unfavorable clinical course of COVID-19 in patients with hematological malignancies came from China and showed mortality of 62% in the small group of 13 hospitalized patients [117]. Along with the worldwide spread of the pandemic, many retrospective reports and meta-analyses confirmed a more severe clinical course of COVID-19 with high morbidity and mortality in patients with hematological malignancies. Incidence, clinical course and risk factors for a severe course of COVID-19 in patients with hematological malignancies are presented in Table 3. 

Mortality among patients with HMs was not only higher than in the non-cancer population but also higher than in patients with solid tumors [139,140]. Sharafeldin et al. reported 15% greater mortality rate from COVID-19 in hospitalized patients with HMs as compared to the non-cancer population. Most authors defined severe clinical course of COVID-19 as the hospitalization with requirement for oxygen or ICU admission. Similarly to the general population, the main risk factors of a severe clinical course were old age and specific comorbidities, such as cardiovascular and renal diseases [141]. Correlation between specific malignancy and the clinical outcome was much more controversial. In some analyses, lymphoid malignancies, mainly CLL and MM, were associated with the highest mortality rate, while in the others, mortality rates were higher in patients with acute myeloid leukemia and myelodysplastic syndrome (Table 3) [124,125,139]. Both CLL and MM are considered as the diseases with the most severe immune defects among all hematological malignancies, involving both humoral and innate immunity. In patients with CLL, mortality due to COVID-19 was about 30% [134,142]. In our analysis of 200 CLL patients, the mortality was 32%, and it was higher in patients receiving anticancer therapy; however, the type of treatment did not influence the outcome (Table 3) [135]. Many authors have reported similar observations, while others have noted the adverse effect of B-cell-depleting therapies on treatment outcomes. In patients with lymphoma, B-cell-depleting therapies such as anti-CD20 monoclonal antibodies [143], BTK inhibitors and CAR-T were associated with unfavorable outcomes of COVID-19 [125,144]. In a cohort of 214 hospitalized lymphoma patients, the mortality rate was 18%, and mortality predictors included active treatment, CAR-T within a year and cardiovascular disease [115]. The CD4+ and CD8+ T-cell counts were significantly lower in patients who died due to COVID-19 within 60 days of infection as compared to survivors [139]. Cook et al. reported a mortality rate of 54.6% in the cohort of 73 MM patients with COVID-19 (Table 3) [132]. According to Ho et al., treatment with anti-CD38 monoclonal antibodies as well as cardiac and pulmonary comorbidities were independent predictors of ICU admission, and cardiac comorbidity was an independent predictor of mortality [118]. Acute myeloid leukemia, on the other hand, usually presents with profound granulocytopenia caused by leukemic infiltration of the bone marrow as well as by chemotherapy. Increased risk of COVID-19 and its severe clinical course was observed in patients undergoing autologous and allogeneic stem cell transplantation, with mortality up to 34%. In some patients, especially with B-cell lymphoid malignancies, persistent SARS-CoV-2 infection was observed lasting for even a few months [122,144,145,146]. The largest cohort of patients with persistent COVID-19 was described by Lee at al., and the authors identified B-cell depletion as the key factor contributing to long-lasting infection. A total of 13.9% of patients showed persistence of a positive PCR result, indicating the detection of SARS-CoV-2 RNA, more than 30 days after the initial positive result. The independent factors predicting prolonged SARS-CoV-2 RNA detection included lymphopenia, treatment with anti-CD20 monoclonal antibodies or SCT within one year [139]. Another issue of clinical significance was delaying of SCT in SARS-CoV-2-positive patients, which could negatively impact prognosis, regardless of clinical symptoms of COVID-19 [147]. Other treatments, such as monoclonal antibodies, CAR-T or high-dose chemotherapy, were also delayed until SARS-CoV-2 tests were negative. Anti-CD20 monoclonal antibodies were not even administered due to concerns about life-threatening infection [138,148,149,150].

In general, the prognosis for COVID-19 was most favorable in patients with chronic myeloproliferative diseases such as chronic myelogenous leukemia and Ph-negative myeloproliferations, as both the underlying disease and the treatment, such as tyrosine kinase inhibitors, alkylating agents or alpha-interferons, do not impair the immune system [125,151,152,153].

In conclusion, SARS-CoV-2 infection caused an increase in the morbidity and mortality of patients with hematological malignancies resulting from COVID-19 and the delayed/deferred anticancer therapy.

### 12.4. COVID-19 in Vaccinated Patients with Hematological Malignancies

The large international multi-institutional COVID-19 and Cancer Consortium (CCC19) registry reported the clinical course of COVID-19 in 1787 patients with current or a prior history of invasive cancers including patients with HMs who developed breakthrough SARS-CoV-2 infection after having received COVID-19 vaccines in comparison to a contemporary unvaccinated population. The analyzed period was from 1 November 2020 to 31 May 2021. In this group, 1656 patients (97%) were unvaccinated, 77 (4%) were partially vaccinated and 54 (3%) were fully vaccinated. Among the fully vaccinated patients who developed COVID-19, 35 (65%) were hospitalized, 10 (19%) were admitted to the ICU or required mechanical ventilation and 7 (13%) died within 30 days. Following Inverse Probability of Treatment Weighting (IPTW), there was no difference in 30-day mortality between the fully vaccinated patients and unvaccinated cohort, with adjusted odds ratio (AOR) 1.08 (95% CI: 0.41–2.82). There were also no differences in ICU/mechanical ventilation or hospitalization rates between the vaccinated and unvaccinated patients (AOR = 1.13 and 1.25, respectively). ICU/mechanical ventilation and hospitalization rates were higher in patients with HMs than in patients with solid cancers (AOR = 2.00 and 2.42, respectively) [111].

The most comprehensive reports on the outcome of COVID-19 in HM patients who received previous vaccination were presented by the European Hematology Association’s Specialized Working Group, Infections in Hematology, which created the EPICOVIDEHA registry to collect data on all adult patients with HMs who developed COVID-19 [129]. Overall, 60.4% of patients had a severe or critical infection, and 66.4% were admitted to the hospital, with 21.3% of hospitalized patients requiring ICU admission. Among patients admitted to the ICU, 62.5% required mechanical ventilation. The overall mortality rate was lower than in the pre-vaccination period (approx. 31%), but it still remained as high as 12.4% with COVID-19 remaining the main or a secondary cause of death for almost all patients. There was no difference in mortality between partially or fully vaccinated patients (15.4% vs. 11.5%) or between patients with a serological response to the vaccine vs. non-responders (13.3% vs. 15.6%). In a multivariable analysis, age was the only factor associated with the risk of death (HR = 1.053, 95% CI: 1.004–1.105). Most deaths were reported among patients with lymphoproliferative disorders (71.4%), and none were reported among patients with acute myeloid leukemia, which is a significant difference compared to the pre-vaccination period [129].

An extension of a previously published study already included a total of 1548 patients, most of whom had lymphoid malignancies (76%) [107]. AML was the most frequent myeloid malignancy (140 cases). At the time of COVID-19 diagnosis, most patients had a clinically stable malignancy (53%), and 23.6% had an active disease. The most common recent cancer therapies prior to COVID-19 were chemoimmunotherapy and targeted therapy, which took place in 36% and 20% of patients, respectively. Nearly 6% of patients received SCT, either allogeneic (4.9%) or autologous (1%), within 6 months before COVID-19, and 0.5% of patients received CAR-T. The majority of patients received two or three vaccine doses before COVID-19 (91%), and most of these were mRNA-based (89%) (Table 2). The majority of patients (55.3%) were considered non-responders to vaccination based on assessment of anti-SARS-CoV-2 spike IgG antibody titers. COVID-19 was severe or critical in 42.7% of patients, which was a significant reduction in comparison to both the pre-vaccination period (63.8%; *p* < 0.001) and the preliminary report [129]. Overall, 53.2% of patients required hospitalization, and among them, 18.1% required ICU admission. As in the preliminary report, the hospitalization and ICU admission rates were significantly lower than in the pre-vaccination era (53.2% vs. 73%; *p* < 0.001% and 9.8% vs. 18.1%; *p* < 0.001, respectively). Fifty-nine percent of patients received COVID-19-specific treatment (monoclonal antibodies: 34.3%, corticosteroids only: 27.1%, antivirals only: 24.1%, antiviral plus monoclonal antibodies: 11.9% and convalescent plasma: 2.5%). Viral genomes were analyzed in 48.6% of patients, and the different Omicron variants were the most frequently detected viral strain (68.7%). The overall 30-day mortality was 9.2%, which was significantly lower than in the pre-vaccination era (approx. 31%). The mortality rate in patients with the Omicron variant was 7.9%, which was comparable to other variants. The primary cause of death was COVID-19 in 67.8% of patients, and a combination of COVID-19 and progressive HM was the cause of death in 27.2% of patients. Importantly, the authors did not find any significant difference in terms of 30-day mortality rate among patients with different HM (*p* = 0.693), which is in contrast to that observed in the pre-vaccination era, when a higher number of fatalities was reported in patients with AML/myelodysplastic syndrome. In the univariable analysis, older age, active HM and severe and critical COVID-19 were significantly associated with mortality. Conversely, patients receiving monoclonal antibodies for COVID-19 had a lower mortality rate. The authors did not find differences in the outcome depending on the number of vaccine doses received. However, a slightly better clinical outcome was noted in patients who received three to four doses. In the multivariable model, older age, active disease, critical COVID-19 and 2–3 comorbidities were correlated with a higher mortality, whereas monoclonal antibody administration, alone or in combination with antivirals, showed a protective effect [129].

EPICOVIDEHA further reported on 102 patients among all 7302 patients with COVID-19, who developed breakthrough COVID-19 after having previously received the fourth COVID-19 vaccine booster [108]. As in previous reports, the majority of patients (84.3%) were diagnosed with lymphoproliferative diseases. Patients with myeloproliferative malignancies constituted solely 14.7%, with half of AML patients having previously undergone allogeneic hematopoietic cell transplantation. Most patients had a clinically stable HM at the time of COVID-19 diagnosis (61.8%), whereas the remaining 37.3% suffered from active disease. More than half of patients (56.9%) received immunochemotherapy as the most recent antineoplastic treatment before COVID-19. COVID-19 remained asymptomatic or mild in most cases (57.8%), whereas 38.2% of cases were defined as severe, and 3.9% were defined as critical and requiring intensive care. The rate of severe/critical infection was significantly lower in comparison to the first preliminary report (42.1% vs. 70%; *p* < 0.001) [106]. The hospital admission rate was 38.2%, with a median hospital stay of 10 days (IQR 7–21). The overall mortality rate was very low, with only four deaths reported (3.9%). In two of those cases, the death was attributable to COVID-19, whereas in the other two, it was due to both COVID-19 and hematological malignancy. Of note, both the hospital admission rate and mortality were significantly lower than previously reported [129]. Only 54.9% of patients received a specific treatment for COVID-19, and 46.4% received monoclonal antibodies while only a few patients received antiviral compounds. Genotyping of VOCs identified Omicron variants in almost all samples, with the most frequently reported sub-variant BA.2 [129].

In the EPICOVIDEHA reports, it was not possible to estimate the actual incidence of SARS-CoV-2 breakthrough infections or the actual number of asymptomatic patients because only patients diagnosed with COVID-19 were included in the registry, and SARS-CoV-2 testing was performed only in symptomatic patients or through screening programs. An attempt to calculate the cumulative risk of breakthrough infections in patients with HM was made by Wang et al. with the same limitations listed previously [109]. In the retrospective cohort study, electronic health records of 514,413 fully vaccinated patients who received two doses of the Pfizer–BioNTech or Moderna mRNA vaccine or a single dose of the Johnson & Johnson (J&J) vaccine from 63 healthcare organizations in the US were analyzed. In this group, there were 5956 patients with HM and 508,457 patients without malignancies reported from December 2020 to October 2021. The cumulative incidence of breakthrough COVID-19 was 13.4% in the entire group, ranging from 11.0% in patients with acute lymphoblastic leukemia to 17.2% and 17.4% in patients with MM and CML, respectively. The risk was 4.5% in patients without malignancies (*p* < 0.001). Patients with breakthrough infections were older, had more comorbidities and received more chemotherapy cycles and targeted cancer therapies. In patients with HM who had breakthrough infection, the hospitalization risk was 37.8% and was significantly higher compared to 2.2% for those who had no breakthrough infection (HR = 34.49; 95). For patients with HM who had breakthrough infection, the overall mortality risk was 5.7% in comparison to 0.8% for those who had no breakthrough infection (HR: 10.25) [109].

Based on the published results, it is worth noting that in the era in which COVID-19 vaccinations were available, as in the pre-vaccination era, the majority of the affected HM patients are diagnosed with lymphoproliferative malignancies during active treatment with immunochemotherapy or targeted agents [108,129]. However, it has to be acknowledged that the proportion of patients with lymphoproliferative diseases has increased in comparison to the first reports, which is consistent with the low immunogenicity of the anti-SARS-CoV-2 vaccine in patients with lymphoid malignancies [108,109,129] and significantly higher immunogenicity in patients suffering from myeloid malignancies [154].

To conclude, the risk for breakthrough COVID-19 is significantly higher in patients with HMs compared to patients without cancer. The ratio of severe/critical COVID-19 is, however, substantially lower in HM patients who received COVID-19 vaccination in comparison to the pre-vaccination era, and it decreases along with the increasing number of administered boosters. The hospitalization rate, especially the need for an ICU admission, and mortality are also significantly lower in HM patients with breakthrough COVID-19. Nevertheless, it has to be acknowledged, that a breakthrough COVID-19 in HM patients may still be associated with non-neglectable mortality. Importantly, the lowest rate of severe/critical COVID-19, hospital admission as well as death is observed in patients who received the second COVID-19 vaccine booster, i.e., the fourth vaccination dose.

Therefore, it needs to be emphasized that vaccination of HM patients is of utmost importance, and a second vaccine booster may be of particular importance to protect patients with hematological malignancies from severe or critical COVID-19.

### 12.5. Metal and Metal Complexes and COVID-19 in Patients with Hematological Malignancies

To the best of our knowledge, there are no reports dedicated to the association between the incidence and outcome of COVID-19, the efficacy of anti-SARS-CoV-2 vaccination and both trace and macro-elements in patients with HM. It may, however, be anticipated that as in a general patient population, changes in the concentrations of certain elements can be more exacerbated in subjects who develop a severe form of COVID-19 compared to those with non-severe COVID-19 and in deceased patients in comparison to survivors, as it was reported by Ścibior et al. [155]. Although not routinely taken into consideration, zinc (Zn) supplementation could potentially be considered in COVID-19 patients, including those with hematological malignancies, as an adjunct therapy. In a randomized, double-blind controlled trial, zinc administration was associated with a decrease in 30-day mortality, ICU admission rate and symptom duration [156]. Additionally, in a meta-analysis by Olczak-Pruc et al. [157], zinc supplementation was associated with lower COVID-19 in-hospital mortality [157]. Importantly, the authors concluded that it was side-effects-free, i.e., not associated with acute renal damage. Unfortunately, the study did not report data for HM patients describing the typical hematological side effects of this trace element. A similar effect to low zinc level was observed for low selenium (Se) concentration in six observational studies across Europe, where non-survivors consistently had lower Se and Zn concentrations than survivors and displayed an elevated Cu/Zn ratio [158].

## 13. Conclusions

In summary, the COVID-19 pandemic reminded that infectious complications are among the most important causes of adverse outcomes in patients with hematological malignancies. It is crucial to realize the risk of infections in patients with particular cancers and during anti-cancer therapy, to make patients and medical staff aware that the pandemic is still ongoing and to remind them about the need for preventive vaccinations and proper hygiene rules in order to effectively protect patients not only against COVID-19 but also other infectious complications.

## Figures and Tables

**Table 1:** Treatment of COVID-19 in patients with hematological malignancies.

Drug	Diagnosis	Number of Patients/Country	Time Period Analyzed	Median/Mean Age (Years)	Mortality Rate (%)	Risk Factors for Severe Clinical Course of COVID-19	Reference
Convalescent plasma	Non-Hodgkin lymphoma	6/Italy	October 2020–May 2021	59.5	16.7	anti-CD20 therapy	Oliva et al. [50]
	Hematological malignancies	13/Sweden	May 2020–March 2021	60.3	38.5	anti-CD20 therapy	Ljungquist et al. [51]
	Hematological malignancies	33/India	May 2020–November 2020	62	45.5	active therapy (24 cases)	Jeyaraman et al. [52]
	Hematological malignancies	7/Italy	March 2020–June 2020	58.6	0	patient after immunochemotherapy/alloHSCT < 1 year (6 cases)	Ferrari et al. [53]
Remdesivir	Hematological malignancies	115/Mexico	December 2021–March 2022	50	8	chemotherapy < 30 days, anti-CD20 therapy, progression of the disease and other	Martin-Onraët et al. [54]
	Hematological malignancies	20/Hungary	December 2020 and May 2021	56	0	anti-CD20 therapy, progression of the disease, stem cell transplantation	Magyari et al. [55]
	Follicular lymphoma, diffuse large B-cell lymphoma	3/USA	No data	53	0	haploidentical bone marrow transplantation, common variable immunodeficiency, chimeric antigen receptor (CAR) T-cell therapy (after fludarabine and cyclophosphamide)	Dioverti et al. [56]
	Acute leukemia, lymphoma, multiple myeloma	32/Czech Republic	December 2020– March 2021	57.7	9	active hematological disease, active therapy	Weinbergerova et al. [57]
Molnupiravir	Hematological malignancies (lymphomas (45%), multiple myelomas (21%) and acute leukaemias or myelodysplastic syndrome (35%))	175/Poland	January 2022–April 2022	56	4	no data	Bołkun et al. [58]
Tixagevimab/cilgavimab	Hematological malignancies	52/USA		62	0	chimeric antigen receptor (CAR) T-cell therapy, alloHSCT, autoHSCT	Stuver et al. [59]

**Table 2:** COVID-19 vaccines used both as primary vaccination and booster approved in European Union countries and their clinical efficacy in cancer patients.

Diagnosis	Number of Patients/ Country	Time Period Analyzed	Median Age; Years (IQR)	Vaccination		Number of Doses	n (%)	Incidence	Clinical Course	n (%)	Death Number (%)	Hospitalization; n (%)/ICU Admission; n (%)	Risk Factors	Reference
Hematological malignancies	113/14 countries	1 January 2021–31 August 2021	66 (58–78)	BioNTech/Pfizer	79 (69.9%)	1	25 (22.1%)	not reported	Asymptomatic	22 (19.5%)	14 (12.4%)	75 (66.4%)/16 (21.3%)	MVA:	Pagano et al. [106]
				Moderna COVE	20 (17.7%)				Mild	12 (10.6%)			higher mortality: age	
				AstraZeneca Oxford	10 (8.8%)	2	88 (77.8%)		Severe	63 (55.8%)				
				CoronaVac/Sinovac	4 (3.5%)				Critical	16 (14.2%)				
Hematological malignancies	1548/26 countries	1 January 2021–10 March 2022	66 (55–75)	BioNTech/Pfizer	1121 (72.4%)	1	129 (8.3%)	not reported	Asymptomatic	306 (19.8%)	30-day: 143 (9.2%)	823 (53.2%)/152 (18.1%)	UVA:	Pagano et al. [107]
				Moderna COVE	256 (72.4%)								higher mortality: older age, active HM disease, presence of two to three comorbidities	
				AstraZeneca Oxford	99 (6.4%)	2 (or J&J)	770 (49.7%)		Mild	604 (39%)			lower mortality: anti-SARS-CoV2-treatment with monoclonal antibodies	
				Sputnik	13 (0.8%)	3	639 (41.3%)		Severe	509 (32.9%)			MVA:	
				J&J (Janssen)	21 (1.4%)								higher mortality: older age, active HM disease, presence of two to three comorbidities	
				CoronaVac/Sinovac	21 (1.4%)	4	10 (0.6%)		Critical	152 (9.8%)			lower mortality: anti-SARS-CoV2-treatment with monoclonal antibodies	
				Sinopharm	17 (1.1%)									
Hematological malignancies	102/12 countries	until August 2022	69 (62–75)	mRNA	101 (99%)	4	102 (100%)	not reported	Asymptomatic	10 (6.9%)	4 (3.9%)	39 (38.2%)/0 (0%)	not reported	Salmanton-García et al. [108]
									Mild	49 (48%)				
				Inactivated	1 (1%)				Severe	39 (38.2%)				
									Critical	4 (3.9%)				
Hematological malignancies	5956 patients with a diagnosis of HM, 508,457 patients without malignancies/US	December 2020–October 2021	Mean + SD: 65.4 ± 15.8	BioNTech/Pfizer	78.2%	2	90%	13.4%	not reported	not reported	Overall mortality risk: 5.7%	37.8%	not reported	Wang et al. [109]
				Moderna COVE	20.8%	1	1%							
				J&J (Janssen)	1.0%									
Hematological malignancies	16/USA	1 December 2020–15 August 2021	35.5 (27.5–44)	mRNA	16 (100.0)	2	15 (3.7%)	not reported	not reported	not reported	1 (6.3%)	5 (31.3%)	not reported	DeVoe et al. [110]
						1	1 (6.3%)							
Solid organ tumors; hematological malignancies	131 (37 HM) out of all 1787 reported/ multinational	1 November 2020–31 May 2021	Fully vaccinated: 65.5 (57.0–72.8); partially vaccinated: 68.0 (58.0–78.0)	BioNTech/Pfizer	77 (58.8%)	not reported	not reported	not reported	not reported	not reported	Fully vaccinated patients; 30-day mortality: 7 (13%)	Fully vaccinated patients: 35 (65%)/10 (19%)	30-day mortality: lymphopenia, the presence of comorbid conditions, worse PS, baseline cancer status (active and progressing versus not active and progressing)	Schmidt et al. [111]
				Moderna COVE	22 (16.8%)								higher ICU/MV and hospitalization rates: lymphopenia, the presence of comorbid conditions, poor ECOG PS, hematologic as opposed to solid cancers	
				J&J (Janssen)	15 (11.6%)									

**Table 3:** Incidence, clinical course and risk factors for severe course of COVID-19 in patients with hematological malignancies.

Diagnosis/Type of Study	Number of Patients/Country	Median Age (Years)	Time Period Analyzed	Hospitalization Rate (%)	Mortality (%)	Risk Factors for Severe Clinical Course of COVID-19	Reference
Hematological malignancies/cohort study	13 (hospitalized)/China	49	23.01.2020–14.02.2020	NA	62	ND	Ho et al. [118]
Hematological malignancies/retrospective study	35/UK	69	11.03.2020–11.05.2020	ND	40 (vs. 14.4 in general population)	age, number of comorbidities, no correlation with active treatment	Aries et al. [119]
Hematological malignancies/retrospective study	34 (hospitalized)/Spain	72	09.03.2020–17.04.2020	NA	33	age, active cancer, AL, MPN/MDS	Martin-Moro et al. [120]
Hematological malignancies/retrospective study	39/Spain	64.7	07.03.2020–07.04.2020	88	35 (vs. 8.5 in general population in Spain)	advanced age (>70), hematological malignancy	Sanchez-Pina et al. [121]
Hematological malignancies/retrospective study	41/Spain	76	08.03.2020–08.04.2020	70	36.6	progressive disease	Infante et al. [122]
Hematological malignancies/retrospective study	367/Spain	64	01.03.2020–15.05.2020	ND	27% (nonSCT: 31%, ASCT: 17%, allo-SCT: 18%)	severe course: hypertension, baseline lymphopenia, CRP > 20 mg/dL mortality: age > 70 years, uncontrolled hematological disease, ECOG 3–4, neutropenia, CRP > 20 mg/dL	Piñana et al. [123]
Hematological malignancies/retrospective study	536 (hospitalized)/Italy	68	25.02.2020–18.05.2020	NA	37	advanced age, progressive disease, AML, NHL, PCN	Passamonti et al. [124]
Hematological malignancies/registry study	697/Spain	72	28.02.2020–25.05.2020	86.5	33	age ≥ 60 years, more than two comorbidities, AML, active MoAbs treatment (Ph’ MPN and HMA were associated with lower mortality)	Garcia-Suarez et al. [125]
Hematological malignancies/retrospective study	740/Turkey	56	11.03.2020–22.06.2020	61.1	13.8 (vs. 6.8 in control group)	hematological malignancies, diagnosis of HCL, AML, MM	Tığlıoğlu et al. [126]
Hematological malignancies	2395 (hospitalized)	ND	01.01.2019–10.03.2021	NA	21.34	ND	Naimi et al. [127]
Hematological malignancies/meta-analysis	3377		01.01.2019–20.08.2020	77	34 (39 in hospitalized)	age > 60 years	Vijenthira et al. [128]
Hematological malignancies/meta-analysis	3801	65	March–December 2020	73	31.2%	diagnosis of AML, MDS, age, active malignancy, chronic cardiac disease, renal impairment, liver disease, ICU stay	Pagano et al. [129]
Lymphoma/prospective observational	177/Spain	70	01.03.2020–30.05. 2020	86.3	34.5	age > 70, heart disease, chronic kidney disease, active lymphoma	Regalado-Artamendi et al. [130]
Lymphoma/retrospective/prospective	237/619/Italy	63	25.02.2020–23.06.2020/23.06.2020–01.02.2021	54.7	19.5	age > 65, male gender, low lymphocyte, low platelet count	Visco et al. [131]
Multiple myeloma/prospective clinical audit	75/UK	73	31.01. 2020–18.05.2020	96	54.6 (vs. 14.5 mortality for COVID-19 in UK)	advanced age	Cook et al. [132]
Chronic lymphocytic leukemia/survey	47/Italy	75	01.04. 2020–15.04.2020	ND	30.4 (vs. 13.4 in general population in Italy and 25.5 in 70–79 years old)	-	Cuneo et al. [133]
Chronic lymphocytic leukemia/retrospective	941/Europe	69	12.2019–03.2021	74.7	27.4 (whole cohort), 38.4 (patients with severe COVID-19)	older age, hypogammaglobulinemia, anticancer treatment, treatment with anti-CD20 antibodies, cardiac failure	Chatzikonstantinou et al. [134]
Chronic lymphocytic leukemia/retrospective study	188/Poland	67.9	25.03.2020–07.03.2021	59	26.6 (38.7 in hospitalized)	age> 65, low PLT, low Hb	Puła et al. [135]
Acute lymphoblastic leukemia/observational study survey	63/Italy		02.2020–04.2021	44.4	11.1		Chiaretti et al. [136]
Acute lymphoblastic leukemia/observational study survey	52/Spain	46.5	01.03.2020–12.01.2021	ND	33	comorbidities	Ribera et al. [137]
Acute myeloid leukemia/observational study	108/Spain	66	13.03.2020–31.05.2020	89.8	43.5	age > 60, gender, active leukemia	Palanques-Pastor et al. [138]

Abbreviations: AL, acute leukemia; MPN, myeloproliferative malignancies; MDS, myelodysplastic syndrome; AML, acute myeloid leukemia; NHL, non-Hodgkin lymphoma; PCN, plasma cell neoplasms; HMA, hypomethylating agents; HCL, hairy cell leukemia; MM, multiple myeloma; ICU, intensive care unit; NA, not applicable; ND, no data.

## Data Availability

All data have been collected from scientific publications available through open access or institutional subscriptions.
